# Effect of Carbon in Fabrication Al-SiC Nanocomposites for Tribological Application

**DOI:** 10.3390/ma10060679

**Published:** 2017-06-21

**Authors:** Bartosz Hekner, Jerzy Myalski, Tomasz Pawlik, Małgorzata Sopicka-Lizer

**Affiliations:** Faculty of Material Engineering and Metallurgy, Silesian University of Technology, Katowice 40-019, Krasińskiego 8, Poland; jerzy.myalski@polsl.pl (J.M.); tomasz.pawlik@polsl.pl (T.P.); Malgorzata.Sopicka-Lizer@polsl.pl (M.S.-L.)

**Keywords:** aluminium matrix composites (AMC), hybrid composites, glassy carbon, carbon nanotubes, powder metallurgy

## Abstract

Aluminium-based hybrid composites are a new class of advanced materials with the potential of satisfying the demands in engineering applications. This paper describes the effects of carbon addition on the formation and properties of AMC with SiC nanoparticles reinforcement. The composites were produced via mechanical alloying followed by hot pressing. Three forms of carbon, graphite (GR), multiwalled carbon nanotubes (CNTs), and, for the first time, glassy carbon (GC), were used for the hybrid composites manufacturing and compared with tribological properties of Al-SiC composite without carbon addition. GC and CNTs enhanced formation of Al-SiC composite particles and resulted in a homogeneous distribution of reinforcing particles. On the other hand, GR addition altered mechanochemical alloying and did not lead to a proper distribution of nanoparticulate SiC reinforcement. Hot pressing technique led to the reaction between Al and carbon as well as SiC particles and caused the formation of Al_4_C_3_ and γ-Al_2_O_3_. The subsistence of carbon particles in the composites altered the predominant wear mechanisms since the wear reduction and the stabilization of the friction coefficient were observed. GC with simultaneous γ-Al_2_O_3_ formation in the hybrid Al-SiC(n)-C composites turned out to be the most effective additive in terms of their tribological behaviour.

## 1. Introduction

Aluminium matrix composites (AMCs) have received considerable interest due to their enhanced properties compared with aluminium alloys in view of improved wear resistance, high strength and stiffness, better creep resistance [1]. Considering tribological applications of AMCs in the automotive industry (brake pads, pistons and piston insert rings, cylinder blocks, etc.) improvement of their wear resistance is essential but the single particulate reinforcement of hard SiC, Al_2_O_3_, TiC, B_4_C, and TiB_2_ micro-range particles [2,3,4,5,6,7] commonly used in those composites suffers from the composite’s hardness increase and an excessive wear of the counterpart. To overcome this problem, incorporation of a solid lubricant (graphite, boron nitride (BN), and glassy carbon) instead of hard particles into AMCs was proposed lately [8,9,10,11,12,13], but some reports point towards deterioration of mechanical properties and/or weakening of the wear resistance if graphite is added as a single reinforcement [14]. Recently, substantial effort has been observed in the development of hybrid composites in order to obtain the synergistic effect of strengthening and lubrication [15,16,17]. Interesting results were observed if carbon nanotubes (CNTs) addition is combined with graphene [18] or, if TiB_2_ particles are used with BN [19] or SiC [20], since formation of lubricating H_3_BO_3_ film was reported recently [21]. This approach, however, requires more advanced manufacturing technology since the homogeneous distribution of both types of reinforcing particles becomes the crucial factor and the contact with liquid Al should be controlled. Powder metallurgy method, among others, allows incorporation of nanosized reinforcement and/or carbon addition in the form of carbon nanotubes (CNTs) [22,23,24], graphene [8], or mutual graphene-CNTs complex additives [18]. This method, combined with mechanochemical processing has been chosen for preparation of the hybrid Al-SiC-C composites in the present report since it allows minimising the contact of molten aluminium with the fine sized carbon items.

The three possible carbon forms were chosen as a solid lubricant for manufacturing the hybrid Al-SiC(n) composites: graphite, glassy carbon and multiwalled CNTs. Both graphite and glassy carbon could act as a solid lubricant. Their particles have similar micro-scale but their thermal, chemical and mechanical properties are different. There are some positive reports on the role of graphite in AMCs: reduction of the coefficient of friction (COF) [9,10] and decrease of wear in aluminium–graphite composites produced by casting with a content of 5 wt % of graphite particles [11]. However, there are some other reports showing no effect of graphite addition, e.g., for Al-SiC composites [16], or showing the key role of a homogeneous distribution of graphite particulates in an aluminium matrix [25]. On the other hand, the improvement of wear resistance was found in the hybrid Al-(Al_2_O_3_-SiC-C) composites where a waste from the coal mine was applied as a source of carbon [26]. The glassy carbon (GC) particles alone were also used to alter the wear behaviour of a eutectic aluminium–silicon alloy produced by the casting method [27]. No report was found in the literature on the hybrid particle reinforced Al-SiC-GC composites, but the latest report [28] demonstrates the positive effect of combination of alumina porous preform with glassy carbon precursor on the wear properties of an Al alloy pressure infiltrated composite.

Choice of CNTs as a lubricant agent in Al-SiC composites could be questionable but the size of multiwalled carbon nanotubes is of the same magnitude as nano SiC particles. Besides, their reverse mechanical properties could have the positive effect on items dispersion during mechanochemical processing of the initial powders. There are reports [28,29,30,31] showing the enhancement of mechanical properties of SiC-multiwalled CNTs hybrid AMCs, however, it requires the formation of strong bonded interface of the particle/matrix. The application of stir-casting [29] or high-energy ball milling [30,31] appeared to be operative in such bonds development.

The aim of this paper is to detect the positive effect and to confront the impact of various carbon forms in the hybrid AMCs via manufacturing the hybrid Al-SiC-C composites and comparing their basic properties and tribological behaviour with the reference ones, produced with the same method. Therefore, technological parameters were kept the same for all specimens but they were not optimised. The paper describes a fabrication method for various carbon (GR, CNTs, or GC)–Al matrix composites with silicon carbide nanoparticles SiC(n) reinforcement. First, the high-energy ball milling was applied for manufacturing Al-SiC(n) composite particles while carbon/graphite particles or nanotubes were believed to modify the milling process. It was expected that high-energy collisions between plastic aluminium particles and hard SiC nanoparticles would result in the size reduction of Al particles with simultaneous encapsulation of nanoparticles because of substantial differences between their surface energy. Consolidation of the resultant powder was carried out via hot uniaxial pressing. Characterization of the successive composites involved XRD studies, hardness measurements, tribological properties and microstructure examination. Finally, the effect of carbon forms on the properties of the hybrid AMCs is discussed.

## 2. Materials and Methods

**Figure 1:**
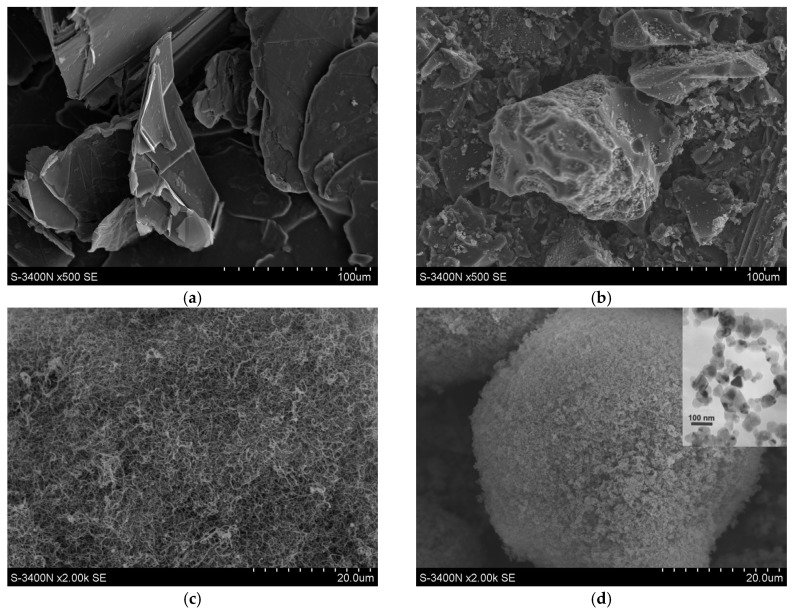
SEM images showing the morphology of: graphite flakes (**a**); glassy carbon particles (**b**); multiwalled carbon nanotubes (**c**); and SiC agglomerates (**d**). Inset: Transmission electron microscopy image showing primary particles in SiC.

The coarse aluminium powder (Avantor Performance Materials Poland S.A., Gliwice, Poland) with a mean particle size in the range of 45–80 μm was chosen for preparation of the composite particles. A silicon carbide nano-powder SiC(n) (EV-C-001 type, Hefei EV NANO Technology Co. Ltd., 99%, Hefei, China) was used as a reinforcing agent. It was in the form of agglomerates with a size in the range of 50–300 µm and was composed of primary particles in the range of 50–100 nm. Three different carbon forms were considered for further studies: graphite, glassy carbon and carbon nanotubes. They have various shapes, diverse mean particle sizes and a different distribution of particle diameter (Figure 1). They have a different crystal form, a dissimilar chemical reactivity and resistance to high energy milling. Graphite powder (P-type, KOH-I-NOOR GRAFIT s.r.o., ash below 6 wt %) and multiwalled carbon nanotubes (C-type, Helix Material Solutions, Richardson, TX, USA) were commercial products. The length of carbon nanotubes was in the range of 1–2 μm. The graphite particles (GR) were in the form of flakes with a diameter in the range of 50–250 μm. Glassy carbon was synthesized at the Silesian University of Technology via pyrolysis of phenol–formaldehyde resins in a vacuum at a temperature of about 1000 °C for 1 h. More details are given elsewhere [32]. Brittle glassy carbon (GC) lumps were initially crushed in a mortar to reduce their diameter below 200 μm.

The composition of powder mixtures is shown in Table 1. The mass fraction was 5 wt % for GR and GC components, whereas it was 1 wt % for CNTs. CNTs mass fraction was reduced to only 1% since a higher amount of this additive considerably affected the subsequent milling. To compensate a various amount of the non-metal reinforcement in Al-SiC and Al-SiC-CNTs composites, the ratio of the metal matrix to the ceramic reinforcement mass fraction was kept constant (5.33) in all tested composites. Furthermore, 4 wt % of stearic acid was added to each mixture in order to prevent welding of metal particles.

The batches were prepared from Al powder, the SiC(n) nanopowder and one form of carbon: CNTs or larger form of carbon particles (GC or GR). Subsequently, 40 g of the weighed out powders with stearic acid and milling balls were placed in a silicon nitride lined jar, closed with a gas-tight cover and flushed out with argon for 30 min. Milling was performed in a planetary ball mill (Fritzch Pulverisette 6 Classic Line, Idar-Oberstein, Germany) for 3 h at 650 rpm, with silicon nitride balls (5 mm), a 4:1 ball-to-powder weight ratio under argon atmosphere. The milling process consisted of a 5 min milling cycle and a 30-min break for cooling. The cycles were repeated 36 times. Then, the gas-tight milling jar with the composite powder was moved to a glove-box filled with argon. Unlocking the jar and storing of the composite powder was carried out in argon atmosphere in order to achieve the controlled passivation of the resultant powder. Next, the composite powder was moved into graphite moulds (30 mm in diameter), placed in the graphite chamber of the furnace, evacuated up to low pressure (0.1 Pa) and hot-pressed (Degussa) at 480 °C for 20 min and then at 700 °C for 15 min under a constant uni-axial pressure of 10 MPa.

The milled composite powders and bulk composites were investigated by Scanning Electron Microscopy (HITACHI S-3400N, Tokyo, Japan) equipped with an energy-dispersive X-ray detector. Transmission Electron Microscopy (HITACHI HD-2300A, Tokyo, Japan) was used for characterization of the milled powders and TEM investigations accompanied with electron diffraction (Selected Area Diffraction Pattern (SADP)) were carried out to identify the microstructure features of the prepared composites.

The density of all bulk specimens was examined with the Archimedes’ method in ethyl alcohol. The theoretical density of composites was calculated according to the mixture rule assuming that the density of Al, SiC, glassy carbon, CNTs and graphite equals 2.72; 3.21; 1.5; 1.3 and 2.2 g·cm^−3^, respectively. These values were used to estimate the composite porosity fraction according to the formula:

P_o_ = 1 − ρ_v_/ρ, (1)

where P_o_ is the volume fraction of open pores, ρ_v_ is the measured density by Archimedes method, and ρ is the calculated density of the composite.

The micro-hardness of the specimens was tested with the Vickers indenter under a load of 20 N for 5 s in Struery Duramin A5 tester (Borken, Germany). The phase analysis (XRD) was performed in a Bruker D8 Discover machine (Poland) with Cu K_α_ radiation source in the 2θ range of 10°–100°. A step size of 0.015° and a scan rate of 0.0075° s^−1^ were applied. The obtained data were then refined with a Rietveld technique.

Dry wear sliding tests were performed in a ball-on-disc equipment (CSM high temperature tribometer with an Instrum X software, Anton Paar, Louxembourg) with friction in air configuration. A load of 10 N and a sliding speed of 0.1 m/s were applied throughout the test. The counter-ball material was made of steel (100Cr6). The tests were carried out over a distance of 100 m at 25 °C. The disc and the ball were weighed before and after the tests (accuracy of 0.001 g) to determine wear loss.

## 3. Results and Discussion

**Figure 2:**
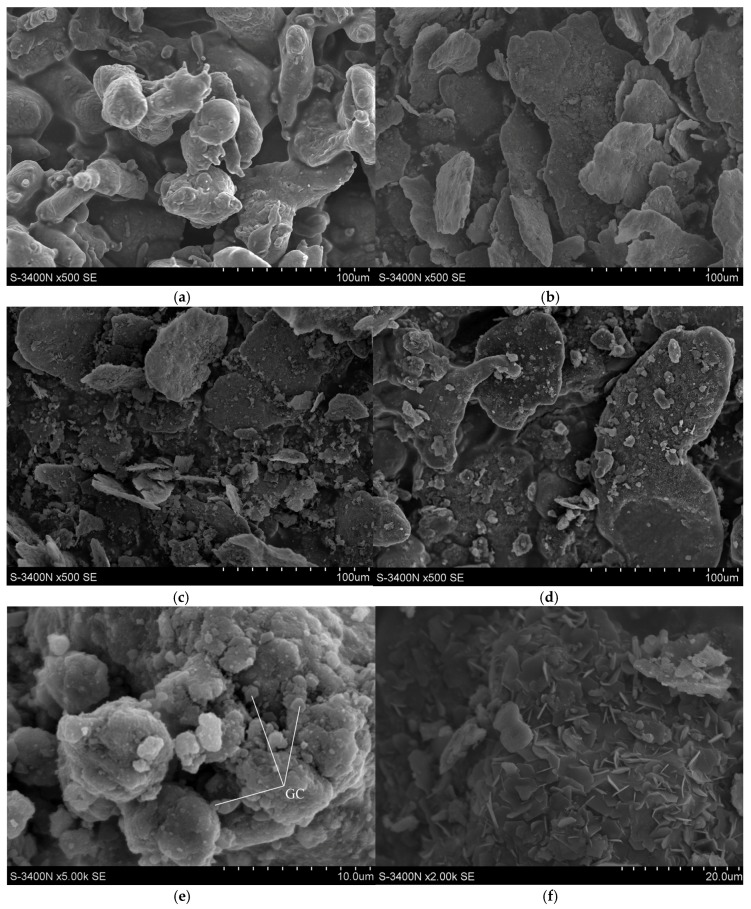
SEM images showing the morphology of: initial Al particles (**a**); Al-SiC(n) composite particles (**b**); Al-SiC(n)-C dual composite powder with GC (**c**,**e**); and Al-SiC(n)-C dual composite powder with GR after mechanochemical processing (**d**,**f**).

The morphology of the different initial carbon particles/nanotubes and the morphology of silicon carbide agglomerates are shown in Figure 1, whereas the morphology of the resultant composite particles is given in Figure 2. Before milling, the graphite particles are in the form of large flakes (Figure 1a); the GC particles demonstrate a typical shape of crumbs as the result of brittle cracking (Figure 1b); the CNTs have a tangled-fibres morphology (Figure 1c); SiC aggregates of nano-particles (Figure 1d); and Al particles (Figure 2a) have an irregular and more or less spheroidal morphology (Figure 1d).

**Figure 3:**
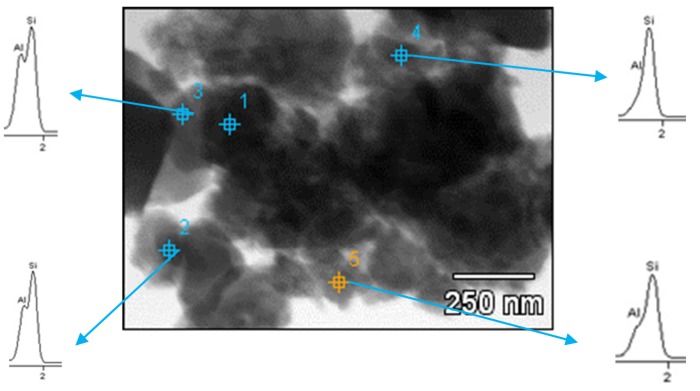
TEM micrograph of Al-SiC(n) composite particles without carbon after high-energy ball milling.

The microscopic studies of the composite powder revealed a dissimilar formation of composite particles under mechanical alloying due to the presence of different carbon forms used. The high-energy milling of the Al-SiC(n) mixture without any carbon addition led to a cold-welding and a severe plastic deformation of ductile Al particles since several particles grew over 100 μm (Figure 2b) with a simultaneous thickness decrease (1–5 µm). SiC agglomerates were not visible at low magnification; they must have been broken into the primary SiC(n) particles and mostly incorporated inside large Al-flakes. TEM studies, however, uncovered an excellent distribution of tiny SiC nanoparticles over the surface of Al particles (Figure 3) since the intensity of Si signal was always higher than that for Al. The Al-SiC solid–solid interfacial energies were found significantly low [33], thus SiC(n) could be easily integrated with Al particles and form hardened composite particles. However, enlargement of the composite particles indicates that SiC nanoparticles alone did not prevent cold-welding of the initial Al particles. The smallest Al-SiC(n) composite particles, nevertheless, were observed for the GC modified powder (Figure 2c,e) and to a lesser extent in CNTs-containing powder (not shown here). For the Al-SiC(n)-GC composition, a large amount of GC particles of various sizes (Figure 2e) were visible among Al-SiC(n) composite particles. They had sharp edges on the contrary to the rounded shape of Al-SiC(n) composite particles and their surface was clean from other types of particles. Some of them were attached to the surface of composite particles. Both GC and CNTs show high solid–solid interfacial energies with aluminium, thus their incorporation into Al particles during the planetary milling would be limited. Contrary to the graphite large flakes, GC particles are brittle and they were broken during high-energy milling. Those broken GC particles or nano-sized CNTs prevented the metal particles from the cold-welding and diminution of their size was observed (Figure 2b,c). Therefore, we can assume that SiC(n) particles in cooperation with both carbon forms (CNTs or GC) acted as solid dispersion agents among Al particles preventing them from welding. A similar role of nano-SiC particles in Al-CNT ball-milled composite powder was observed by Wang et al. [31] and Kwon et al. [34]. Carbon nanotubes, however, were not found among the studied composite powder particles, or in final composites. They could have been broken into pieces of a smaller length and were not recognized during microscopic studies because their diameter was smaller than other microstructure items. Up to now, different behaviour of CNTs after ball-milling with Al alloys have been reported. Esawi et al. [35] did not find intensive breakage of CNTs after the process but Wang et al. [31] and Wu et al. [36] experienced a severe breaking of CNTs from the initial length of 5–20 μm to less than 1 μm after 3 h of milling. They also observed gradually burying of CNTs beneath the Al powder surfaces in the course of the longer milling.

The addition of GR led to the formation of the largest aluminium particles (200–350 µm) with a rounded shape and high thickness (5–10 µm) (Figure 2d,f). Their surface was decorated with small (2–3 μm) hexagonal graphite platelets. Besides, some SiC agglomerates have not been broken and they remained non-bonded to Al matrix particles (not shown here).

**Figure 4:**
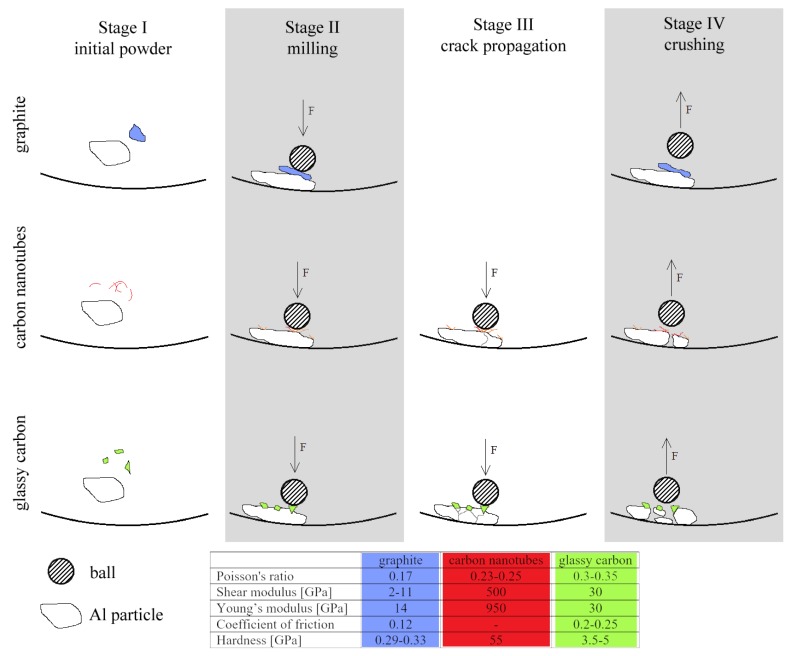
The model of different types of carbon behaviour during milling with aluminium particles.

In mechanical alloying, two competing processes are involved: cold welding and fracturing of particles as a result of the collisions between the powder particles and the milling balls. The complex morphological and microstructural transformations during the colliding milling in the impact-friction contact depend on the relative impact velocity which could be altered by the elastic properties and COF of the particles involved [37]. The elastic module of tested forms of carbon substantially deviates from 4–27 GPa for anisotropic graphite to 270–950 GPa for CNTs and about 32 GPa for isotropic GC [38]. It is possible that the lubricant effect of GR particles and different elastic properties of various forms of carbon modified the course of milling thus only a limited number of Al-SiC composite particles were produced or a very limited number of SiC(n) entered Al particles in the presence of graphite particles. Consequently, cold welding of aluminium particles prevailed over their breaking. The differences in the milling behaviour with the addition of different types of carbon are schematically illustrated in Figure 4. Moreover, the latest reports show that the graphite lubrication effect during high-energy milling could be overcame by prolonged milling with high energy [39] or by metallization of graphite particles before milling with Al powder [40].

The attempts to further hot-press the resultant Al-SiC(n)-GR powder in the semi-liquid state failed because of the significant metal outflow during the process. It could happen because only a small number of the Al-SiC(n) composite particles, resistant to melting at 700 °C at applied pressure, formed at a graphite presence. On the contrary, the large welded Al particles with high surface energy could easily aggregate because the very high wetting angle of liquid Al on graphite (157° at 1100 °C) [41] did not prevent it. Therefore, they melt and they promote a metal outflow. Consequently, this composition was abandoned for further studies.

The physical characterization of the resultant bulk composites is given in Table 2. Density measurements showed a small porosity of the resultant composite specimens and the clear influence of carbon presence on a density reduction is visible. However, if the porosity of the resultant composites is compared, it can be found that the strongest effect of carbon presence occurred in the CNTs reinforced composites. The similar effect of CNTs in Al-SiC(n) composites was found by others [42]. However, the longer milling time was applied, the smaller density drop of the composite was observed. The hardness of the resultant Al-SiC-CNT composites was 10 times higher as compared to aluminium (0.15 GPa) while that of glassy carbon reinforced composites was only five times higher.

**Figure 5:**
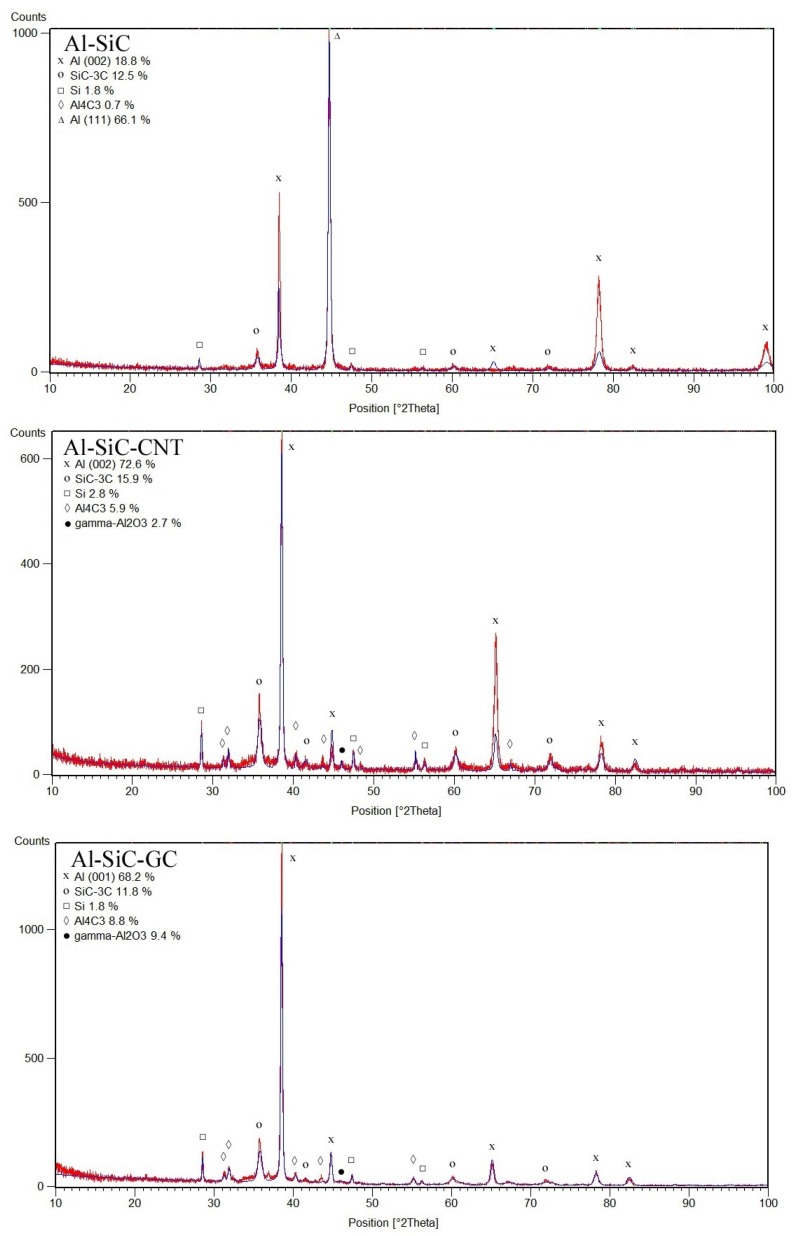
XRD results showing the phase assemblage of the hot pressed composites.

XRD results in Figure 5 do not show the precise quantitative phase composition of the resultant composites because the amorphous phases (carbon, silica scales on nano SiC particles) are not detected by this technique, also a noteworthy aluminium texturization could considerably change the calculated Al weight fraction. Notably, an aluminium amount in an Al-SiC-GC specimen, calculated from the [001] texture, could be underestimated. Consequently, estimation of error is not possible, thus we simply present the as-calculated data from Rietveld refinement (Figure 5) with a discussion below. Apart from an uncertain amount of aluminium, however, it does allow a comparison between other phases and systems tested, as well as an assessment of the occurred reactions.

First of all the XRD results show the possibility of various reactions in the system since aluminium carbide and silicon were found in all specimens tested after the semi-liquid process (Figure 5). The presence of silicon was reported earlier [43] as a result of the direct reaction between liquid aluminium and silicon carbide particles at high temperature, in accordance with the following reaction:

4 Al (l) + 3 SiC → Al_4_C_3_ + 3 Si, (2)

However, Reaction (2) shows the positive free Gibbs energy change of this reaction over 200 °C. Several later reports were aimed at searching the ways of avoiding decomposition of SiC particles in liquid aluminium [44,45]. Some other reports did not depict formation of silicon [16,42]. The simultaneous decline of Al and SiC amount in the tested composite could be also related to additional reaction between molten Al and silica scales on SiC nanoparticles according to the reaction with a negative free Gibbs energy change at 700 °C:

2 Al (l) + SiO_2_ + CO → Al_2_O_3_ + Si + C (3)

A small amount of CO could be derived from the graphite mould and oxygen traces and/or as a result of silica scales reduction by the graphite mould according to the reaction:

SiO_2_ + 2 C = Si + 2 CO (4)

Alumina was only not detected in Al-SiC composites because its amount was below the detection scale or it could be present in a form of amorphous scales on SiC nanoparticles.

The carbon addition obviously modifies reactions during semi-liquid hot-pressing since a higher amount of aluminium carbide is present apart from an increasing amount of aluminium oxide (Figure 5). These reactions are accompanied by a further decreasing of aluminium amount thus a direct reaction of liquid Al with carbon particles can occur:

4 Al (l) + 3 C (s) → Al_4_C_3_ (5)

In specimens with CNTs addition, no decomposition of SiC(n) particles was noticed apart from the reduction of their oxide scales (Si increase) as the result of Reactions (3) and (4). Besides, thermodynamic assessment shows that increase of carbon content in the Al-Si-C-O system improves SiC stability. Hence, aluminium carbide and oxide were formed at the expense of liquid aluminium: Reactions (3) and (5). Previous work [42] has already reported that the Al_4_C_3_ formation was enhanced in CNT presence and it was dependent on the milling time. The resultant phase composition of the reference composites Al-SiC(n) and those modified with CNTs is in a good agreement with high hardness of those specimens.

**Figure 6:**
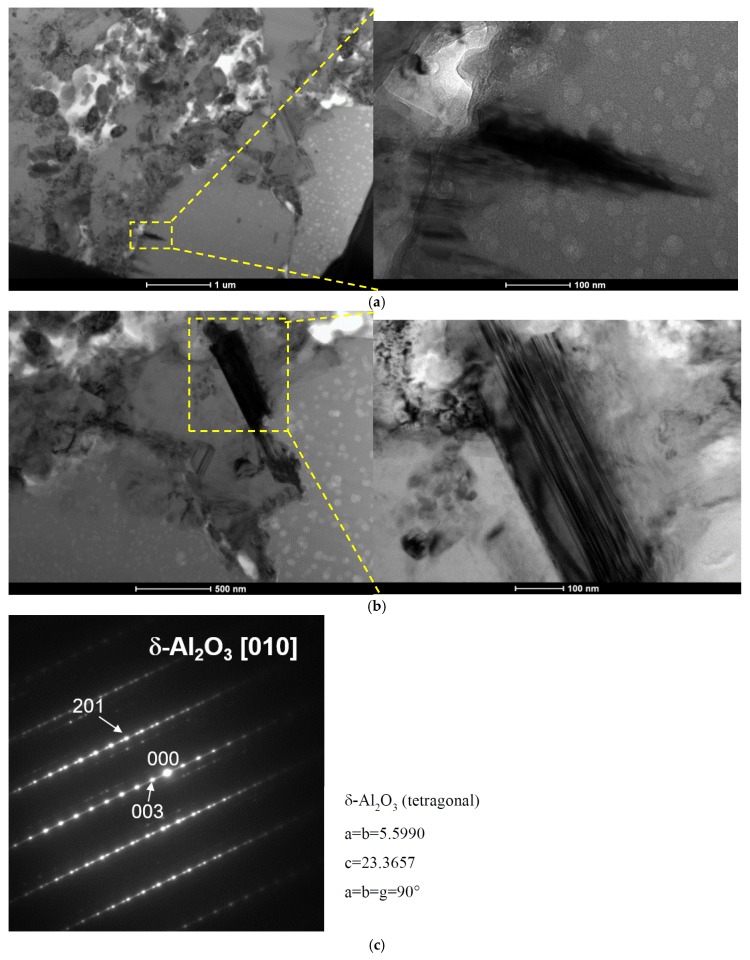
TEM images of δ-Al_2_O_3_ phases. Bright field images (**a**,**b**) and identification of phases (**c**).

The effect of GC on Reaction (5) is more enhanced if compared with CNTs since 50% increase of Al_4_C_3_ was noticed and it was obviously related to larger mass fraction of GC in comparison to CNTs in tested composites. Simultaneous reduction of an Al amount and increase of γ-Al_2_O_3_ suggests the considerable contribution of Reaction (3) in the formation the phase composition of specimens. TEM studies revealed formation of both γ,δ-Al_2_O_3_ transition phases since δ-Al_2_O_3_ was found in the form of needles on the Al matrix- GC interface (Figure 6). The reason of lower than expected SiC amount is not clear and more complicated gas phase reactions could occur among Al_2_O (g), Al (g), SiO (g) in presence of CO (g) abundance. It is interesting to note that Al-SiC(n)-GC composites show meaningfully lower hardness by comparison to the reference ones and those with CNTs reinforcement. All changes of the phase composition (decomposition of SiC hard particles, formation of Al_2_O_3_ transitions) and GC presence could be responsible for a lower hardness of those composites (Table 2) but noteworthy occurrence of soft γ,δ-Al_2_O_3_ seems to be crucial in their tribological behaviour. Formation of soft alumina was not previously reported in similar systems but GC particles were not used in fabrication of Al-SiC(n) composites or amount of SiC(n) was negligible (1 vol %) [42] or SiC particles were large (30–50 μm) [15,16,43], thus their surface area and related silica scales were relatively low. On the other hand, the lubricated effect in TiB_2_/Al composites were found because of oxidation of hard TiB_2_ particles to soft B_2_O_3_ [21].

**Figure 7:**
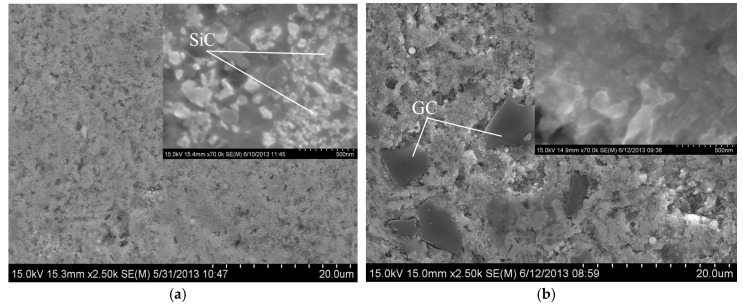
SEM images showing the surface of: the Al-SiC-CNT composite (**a**); and Al-SiC-GC composite (**b**). Insets: Detailed view of microstructure.

The second effect related to the carbon presence shows changes in Al preferred crystallographic orientation of grains as a result of crystallization after semi-liquid hot-pressing. Nevertheless, the reason for this effect is not clear; a possible explanation involves the distinct powder particles morphology before pressing. SEM studies show differences in the microstructure of the resultant composites (Figure 7). SiC(n) particles are homogeneously distributed in the Al matrix in all composites, CNTs were not found and only large GC particles were visible. Small GC particles could have been consumed by Reaction (5) and led to increase of Al_4_C_3_. All those reactions could considerably change the resultant microstructure of composites and affect their wear behaviour.

**Figure 8:**
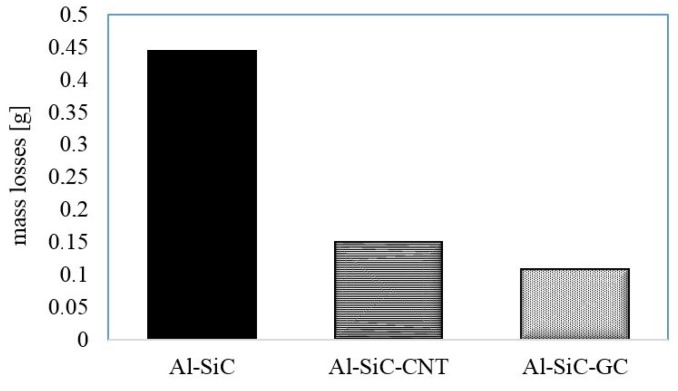
Mass loss of the composites after the room-temperature test.

**Figure 9:**
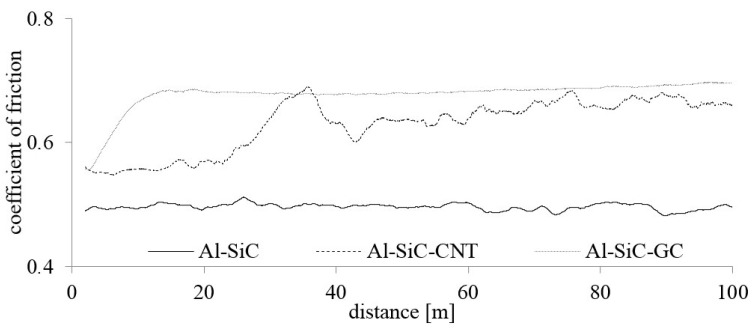
Results of the room-temperature friction tests.

The results of material behaviour in friction conditions at 25 °C are shown in Figure 8 and Figure 9. The comparison of the COF and the wear rate at room temperature revealed the impact of the different carbon sources. The highest mass losses were observed in the carbon-free reference specimen, whereas carbon modified composites showed roughly three times higher wear resistance (Figure 8). The effect of GC modification was slightly stronger if compared with CNTs outcome but a lower mass fraction of CNTs could be a possible reason. The noteworthy effects are also visible if friction behaviour is considered. The reference material showed quite a stable COF on the level of about 0.5, which did not significantly change during the friction test (Figure 9). However, the highest and the most stable COF was observed for a GC modified composite. Indeed, after the effect of the initial stage behaviour, the COF of those composites stabilized at the value of 0.68. Contrarily, the addition of CNTs led to the deterioration of the friction properties since a higher fluctuation of the COF was observed over the tested distance with an average value just below that of the composite with GC.

The similarity of both the CNTs and GC composite’s wear behaviour as well as their phase composition suggests the CNTs were not totally damaged in the manufacturing process. First, the GC particles and CNTs could have been mechanically damaged during mechanochemical processing and/or they could have reacted with molten aluminium as evidenced by the formation of Al_4_C_3_ (Figure 5). Specifically, the fine GC particles could have been consumed in Reaction (5) and only the largest ones were left (Figure 7b). Similarly, CNTs could react with molten Al in their open tips, as it was reported earlier [34,42]. Therefore, their length could have been further reduced after hot pressing. Consequently, a substantial difference between the different carbon modified composites investigated is, first, linked to the size and morphology of the carbon additive: the largest GC particles are preserved after composite processing but the CNTs are chopped and they are hardly seen under the microscopic examination.

As a consequence of an important difference in the size and morphology of carbon additives, the improvement of wear behaviour was more pronounced in GC modified composites but it requires a higher mass fraction of carbon. Improvement of wear behaviour refers to the formation of tensile stresses around the CNTs and GC particles because of a large difference in the thermal expansion coefficient among carbon forms (about 2 × 10^−6^ °C^−1^), and aluminium (23 × 10^−6^ °C^−1^). Formation of soft γ-Al_2_O_3_ with ability or alteration of the wear performance is more enhanced in Al-SiC(n)-GC specimens because of its higher mass fraction. Finally, incorporation of GC particles into AMC-C hybrid composites was undemanding by comparison to graphite or CNTs because of their influence on porosity formation. On the other hand, Al_4_C_3_ formation should be avoided and optimization of PM technology by tailoring temperature and the time of hot pressing is necessary. Those results support an assumption that glassy carbon modification of Al-SiC(n) composites could be more effective and also efficient if availability and simplicity of manufacturing were considered.

## 4. Conclusions

In this study, Al-SiC(n)-C composites with various carbon forms, namely graphite, CNTs and glassy carbon, were fabricating by application of mechanical alloying for manufacturing composite particles and subsequent semi-solid composite powder processing. The effects of type of carbon on the morphology of the Al-SiC(n)-C powder particles after mechanical alloying and composite microstructure, density, hardness, phase composition and wear behaviour after hot-press were analysed.

The mechanical alloying was successful in homogeneous distribution of reinforcing SiC nanoparticles and their enclosure inside Al particles. All tested carbon forms modified the efficacy of mechanical alloying and the subsequent ability of the powder for densification. Because of a low solid–solid interfacial energy of Al-SiC, nano SiC particles were dispersed inside large aluminium flakes. Not all carbon particles were able to enter Al particles because of a high interfacial energy of Al-C and they remained among metal particles or they were found on their surface. Addition of CNTs or GC prevented Al particles from cold-welding and thin hardened composite particles were produced with broken GC particles among them. CNTs must have been chopped during mechanical alloying since they were not visible under microscopic studies. In contrary, graphite particles appeared to be inefficient in formation of Al-based composite powder during high-energy ball milling since they did not prevent aluminium cold-welding and SiC nanoparticles dispersion. Carbon presence in Al-SiC(n) powder altered densification during hot-press in the growing order: GR, CNTs and GC. Graphite-modified composites experienced metal outflow during hot-press, were porous and they were excluded from the further studies. Presence of both carbon forms led to noteworthy consumption of aluminium and formation of undesirable Al_4_C_3_ during densification. This reaction, however, was accompanied by formation of γ,δ-Al_2_O_3_ as a result of reduction of silica scales on SiC particles by aluminium in the presence of CO. A higher amount of soft alumina reduced hardness of composites and it was responsible for beneficial wear behaviour of GC, and, to some extent, CNTs modified Al-SiC(n) hybrid composites. The presence of GC stabilizes the COF on the level near 0.6. Detailed tribological properties and microstructure studies of those composites after the wear test at ambient and elevated temperature can be found elsewhere [46]. Glassy carbon modification seems to be more perspective and efficient in further research if formation of Al_4_C_3_ is limited during densification stage.

## Figures and Tables

**Table 1:** The chemical compositions of manufactured composites.

Composites	Compounds				
	Aluminium (wt %)	Silicon Carbide (wt %)	Carbon Nanotubes (wt %)	Glass Carbon (wt %)	Graphite (wt %)
Al-SiC	84.2	15.8	-	-	-
Al-SiC-NT	83.4	15.6	1	-	-
Al-SiC-GC	80	15	-	5	-
Al-SiC-GR	80	15	-	-	5

**Table 2:** The physical characterization of resultant composites.

Specimen	Density (g/cm^3^)	Porosity Fraction (%)	Hardness (GPa)
Al-SiC	2.76	1.0	1.57
Al-SiC-CNT	2.50	1.0	1.60
Al-SiC-GC	2.64	3.0	0.75
